# The Nottingham Ischaemic Cardiovascular Magnetic Resonance resource (NotIs CMR): a prospective paired clinical and imaging scar database—protocol

**DOI:** 10.1186/s12968-023-00978-1

**Published:** 2023-11-27

**Authors:** Nikesh Jathanna, Kevin Strachan, Bara Erhayiem, Hazlyna Kamaruddin, Peter Swoboda, Dorothee Auer, Xin Chen, Shahnaz Jamil-Copley

**Affiliations:** 1 Department of Cardiology, Nottingham University Hospitals NHS Trust, Nottingham, UK; 2 Queen’s Medical Centre, NIHR Nottingham Biomedical Research Centre, University of Nottingham, Nottingham, UK; 3 Leeds Institute of Cardiovascular and Metabolic Medicine, University of Leeds, Leeds, UK; 4 Department of Computer Science, University of Nottingham, Nottingham, UK

**Keywords:** Ischaemic heart disease, Myocardial fibrosis, Artificial intelligence, Late gadolinium enhancement cardiac magnetic resonance imaging, Curated database, Protocol

## Abstract

**Introduction:**

Research utilising artificial intelligence (AI) and cardiovascular magnetic resonance (CMR) is rapidly evolving with various objectives, however AI model development, generalisation and performance may be hindered by availability of robust training datasets including contrast enhanced images.

**Methods:**

NotIs CMR is a large UK, prospective, multicentre, observational cohort study to guide the development of a biventricular AI scar model. Patients with ischaemic heart disease undergoing clinically indicated contrast-enhanced cardiac magnetic resonance imaging will be recruited at Nottingham University Hospitals NHS Trust and Mid-Yorkshire Hospital NHS Trust. Baseline assessment will include cardiac magnetic resonance imaging, demographic data, medical history, electrocardiographic and serum biomarkers. Participants will undergo monitoring for a minimum of 5 years to document any major cardiovascular adverse events. The main objectives include (1) AI training, validation and testing to improve the performance, applicability and adaptability of an AI biventricular scar segmentation model being developed by the authors and (2) develop a curated, disease-specific imaging database to support future research and collaborations and, (3) to explore associations in clinical outcome for future risk prediction modelling studies.

**Conclusion:**

NotIs CMR will collect and curate disease-specific, paired imaging and clinical datasets to develop an AI biventricular scar model whilst providing a database to support future research and collaboration in Artificial Intelligence and ischaemic heart disease.

**Supplementary Information:**

The online version contains supplementary material available at 10.1186/s12968-023-00978-1.

## Background

Digital technologies, particularly artificial intelligence (AI), are advancing at a significant rate with growing interest and an increase in their application and deployment in clinical medicine. Within radiology, oncology and cardiology use of AI has gained significant momentum due to the data-rich nature of medical imaging.

Cardiovascular magnetic resonance (CMR) has a significant, and established, role in international guidelines [1, 2]. In particular, this modality demonstrates the specific benefit of tissue characterisation with late gadolinium enhanced (LGE) sequences providing vital information in ischaemic heart disease. An area of particular interest is the potential of accurate and detailed characterisation of scar improving the mechanistic understanding of arrhythmogenic scar, risk stratification and delivery of targeted ablation therapy in the management of complex arrhythmias [3].

Several AI models have utilised LGE-CMR datasets for clinical challenges, including diagnostics, myocardial/scar segmentation and prediction modelling, with promising results [4]. However, despite the requirement of large datasets for the development and training of high-performance AI models, we identified that only small numbers of LGE-CMR studies are often used for assessing AI segmentation. This risks overfitting and/or poor generalisability of models [5]. These limitations present significant barriers to advancement, and the ultimate clinical application, of AI models [6–9].

Several open access datasets already exist. However, low numbers and limited sequences restrict their utilisation for AI training [10]. Projects such as euCANshare aim to catalogue and harmonise datasets for optimised identification and collaboration but are currently limited in numbers [11]. The largest LGE-CMR database available for data transfer contains 150 patients (1/3 “normal” and 2/3 “acute myocardial infarction”) with some paired clinical information but no follow-up or outcome data[12]. As such, there is a need for large, paired, clinical and imaging datasets that are accessible to researchers for AI modelling.

Further benefit may be gained by the inclusion of a variety of scar visualisation sequences utilised in clinical practice as well as scanner variations such as manufacturers and field strength with supplementary participant demographics. This is particularly true for patients with implantable cardioverter defibrillators who are often excluded from research studies due to significant device artefact affecting image quality.

Insight obtained from our initial experience of developing an AI segmentation model using 500 2D LGE-CMR scans from two UK sites has highlighted the limitations posed by retrospective data collection including limited access to detailed clinical outcomes and acquisition sequences. Nonetheless, this retrospective, disease-specific, multi-vendor, multi-sequence LGE-CMR database has been critical for the preliminary AI development utilising the most commonly available clinical LGE-CMR manufacturers and acquisition sequences.

Prospectively we aim to develop a robust, paired, clinical and curated LGE-CMR imaging database including multiple manufacturers and a variety of acquisition sequences to broaden AI model training exposure, model refinement, validation and testing. The subsequent disease-specific curated data aims to support future collaborative research projects and clinical outcome prediction modelling.

## Methods and design

### Objective

A prospective disease-specific (ischaemic heart disease), paired, multi-vendor, LGE-CMR imaging and clinical database, “NotIs CMR”, will be created with the following objectives:

1. To broaden and improve the performance, applicability and adaptability of our AI segmentation model using varied CMR sequences available in routine clinical practice.
2. Develop a curated database, available via application, to support broader research opportunities and collaboration.
3. Allow examination of associations between clinical data, imaging data and clinical outcomes.

### Study design

**Fig. 1:**
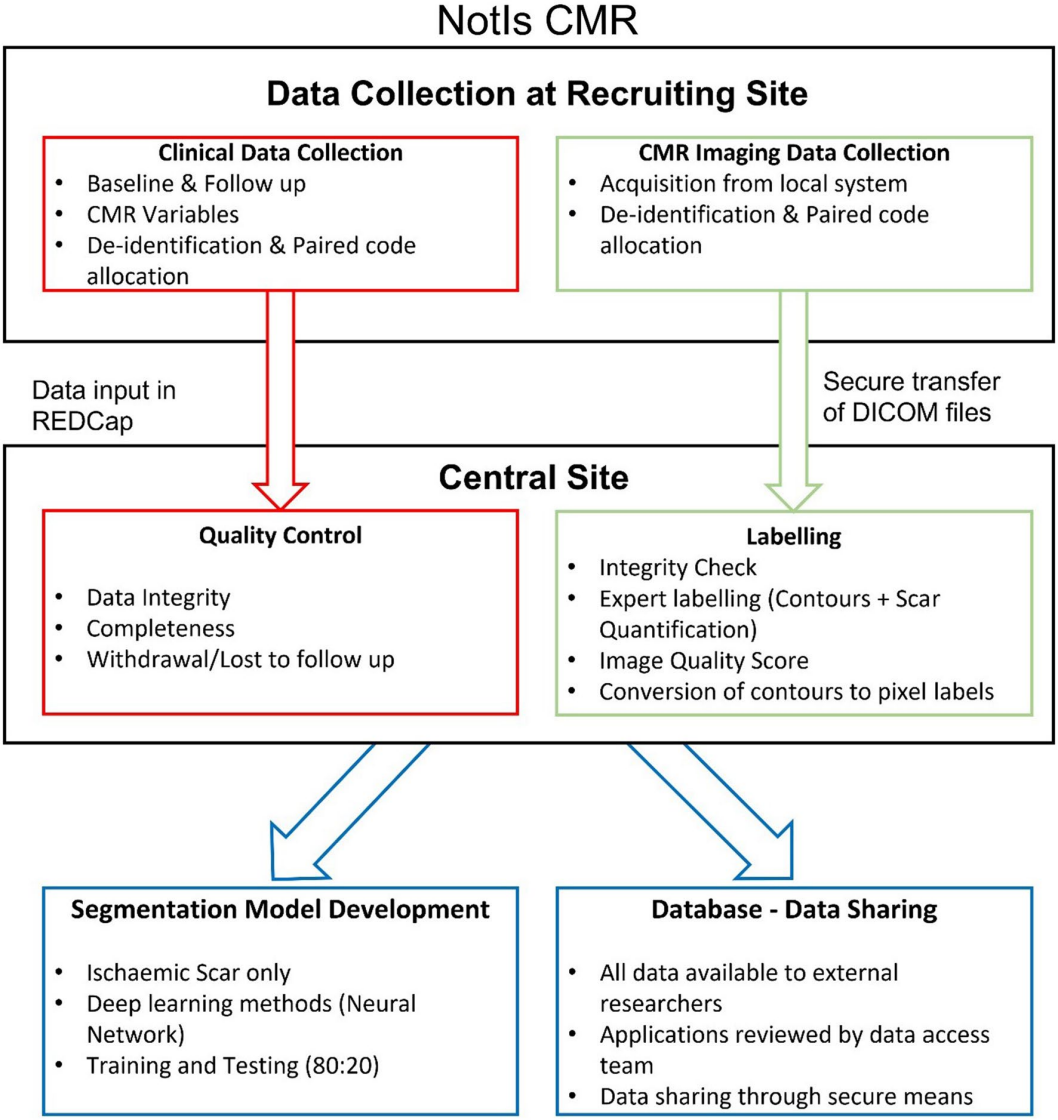
Proposed study workflow. *CMR* Cardiac Magnetic Resonance Imaging

A non-randomised, observational, multicentre resource for the recruitment of routinely acquired LGE-CMR scans in patients with ischaemic scar, including those with implanted cardiac devices e.g. implantable cardioverter defibrillators (Fig. 1).

### Target population

Prospective recruitment will include all adults above 18 yrs of age with no contraindication to LGE-CMR, attending for clinically indicated scans with presumed or confirmed ischaemic aetiology. “All comers” will be approached for full inclusivity, to reflect the local demographics of the disease condition and to support broad recruitment.

### Recruitment and consent

Image segmentation is a key component of this study. As little guidance exists on sample-size calculations in AI model development we aim to collect a large dataset of disease specific scans for this purpose [13].

Having developed our preliminary AI scar model utilising 500 retrospective LGE-CMR scans, the NotIs CMR study will recruit a further 500 participants prospectively based primarily on the feasibility of the two recruiting sites. We will use cross validation and sub-sampling methods to investigate the effects of sample size on our AI model.

Patients, with confirmed or presumed ischaemic heart disease, will be identified either at the time of referral for CMR, at the time of CMR attendance or shortly after CMR acquisition.

Exclusion criteria include clinical contraindication to CMR or inability to provide informed consent. Subsequent absence of ischaemic scar on CMR in those recruited will not result in exclusion from the data resource but will not count towards the ischaemic target required for the study.

Recruitment will complete once 500 scans with ischaemic scar have been collected.

Consent will be obtained from individuals explicitly acknowledging the use of their de-identified data in prospective AI and computational modelling projects. All participants have the right to withdraw from the study at any point.

### Data collection

Data including baseline demographics, risk factors, medical history, routinely reported clinical CMR parameters, standard clinical blood tests, electrocardiograms and clinical outcomes will be stored on the research electronic data capture (REDCap) database.

Imaging data will be obtained from the clinical CMR reports. Follow-up data will be collected from primary and secondary care medical case notes at predefined times up to 5 years post enrolment. This will include major adverse cardiac events (MACE), ventricular arrhythmias, cardiac procedures and death (Tables 1, 2). Definitions are included in the Additional file 1.

**Table 1:** Clinical baseline and outcome variables

Type	Variable
Baseline	Age, gender
	Ethnicity
	Height, weight
	Indication for clinical test
	Resting systolic blood pressure
	Cardiac Risk factors: smoking, diabetes, hypertension, hypercholesterolaemia
	Previous myocardial infarction and intervention
	Implantable Cardiac Device
	Previous Cardiac Surgery
	NYHA and CCS Class
	Previous Arrhythmic Events
	Other respiratory/renal disease
	ECG
	Current Medications
	Blood results
Outcome	All-cause mortality, myocardial infarction, heart failure admission, stroke, arrhythmic events, cardiac device implantation

*NYHA* New York Heart Association*, CCS* Canadian Cardiovascular Score*, ECG* electrocardiogram

**Table 2:** Table of Scheduled Events

Procedures	Visits							
	Screen	Baseline	6 months	12 months	2 years	3 years	4 years	5 years
Informed consent	x							
Demographics		x						
Medical history		x						
CMR data		x						
Medications review		x	x	x	x	x	x	x
Review of Cardiac outcomes			x	x	x	x	x	x

*CMR* Cardiac Magnetic Resonance Imaging

We acknowledge the limitations of robust capture of ventricular arrhythmias in a study where only a proportion of participants will have implanted cardiac devices for monitoring however to maximise data capture, participants will provide consent allowing direct contact with them should further data clarification become necessary.

### Diagnosis

The primary diagnosis will be confirmed only after acquisition of CMR sequences and clinical reporting. Ischaemic heart disease will be defined as either (a) presence of ischaemic-pattern LGE in the absence of any other significant cardiac findings OR (b) alternative imaging confirming the presence of atherosclerotic disease or a history of previous coronary artery bypass grafting/coronary artery intervention, in the absence of ischaemic LGE pattern on CMR.

Whilst all patients recruited will have their data collected and stored, only those with confirmation of ischaemic heart disease on the subsequent CMR results will be included in the specific ischaemic database. The subsequent “normal and non-ischaemic” scans will still be utilised as the “control group” for ultimate AI performance testing.

### CMR acquisition

Local clinical protocols will be utilised to acquire CMR imaging data. LGE images will be typically acquired 10–20 min after a single bolus of 0.1mmol/kg gadobutrol (Gadovist^®^, Bayer Pharma, Berlin, Germany). Examples of LGE protocol parameters can be found in Table 3. Collected data from the clinical reports and image headers will include the specific LGE protocols, slice thickness, spatial resolution and number of slices (Table 4).

**Table 3:** Example of late gadolinium enhanced magnetic resonance protocols utilised at sites

	Site 1 (2D LGE)	Site 1 (3D WHI)	Site 2 (2D LGE)
Scanner	Philips Ingenia	Philips Ingenia	Siemens Aera
Contrast Agent	Gadovist	Gadovist	Gadovist
Field Strength (Tesla)	1.5	1.5	1.5
Inversion pulse	Every 2nd heartbeat	Every heartbeat	Every 2nd heartbeat
PSIR	Yes	No	Yes
Repetition time (ms)	6.1	4.4	6.3
Echo time (ms)	3	2	1.37
Flip angle (degrees)	25	15	50
Slice Gap (mm)	0	0	2
Slice thickness (mm)	10	1	8

*3D WHI* 3D Whole heart Imaging*.* LGE late gadolinium enhanced imaging. PSIR Phase Sensitive Inversion Recovery

**Table 4:** CMR variables ascertained from clinical imaging reports and imaging data

LGE CMR	Variable
	Diagnosis
	Scanner manufacturer and magnet strength
	Slice thickness, compression and acquisition settings
	Image Quality
	Ejection Fraction & myocardial volumes
	Scar presence, location, amount

*CMR* Cardiac Magnetic Resonance Imaging

In patients with implantable devices, local safety policies, in keeping with national and international guidelines, will be followed. These require the completion of MRI safety checklists and questionnaires with close communication between referring clinicians, MRI radiographers and device physiologists prior to, and on the day of, the patient’s scan. Considerations include device type, device dependency and MRI compatibility. During imaging the patient will be monitored closely, with appropriate device programming pre- and post-scan for safety.

To improve image quality in the presence of significant device related artefact efforts will concentrate on patient arm positioning and breath-holding techniques rather than the use of alternative sequences (e.g. wideband).

### Cardiac outcome data

Clinical outcomes will include death (including aetiology), ventricular arrhythmic events, ischaemic events, heart failure admissions and cardiac device implantation (Additional file 1).

### Anonymisation and storage

All images will undergo de-identification at the recruiting sites using dedicated software to remove original patient identifiers including name, medical record number and study acquisition date. De-identified images will be transferred to the central site for analysis and storage via a secure method. Each patient will be allocated a subject number to allow all data to be link-anonymised to their number so no personal identifiable data is stored. Corresponding images and collected clinical information will be linked to the subject number for pairing. Both recruitment sites will retain a cross-referenced list of research participants and their subject numbers stored securely in accordance with GDPR and local policies. This will not be available within the NotIs CMR database nor will it be available to future researchers or those undertaking analysis.

### Image curation

#### Anatomical labelling

**Fig. 2:**
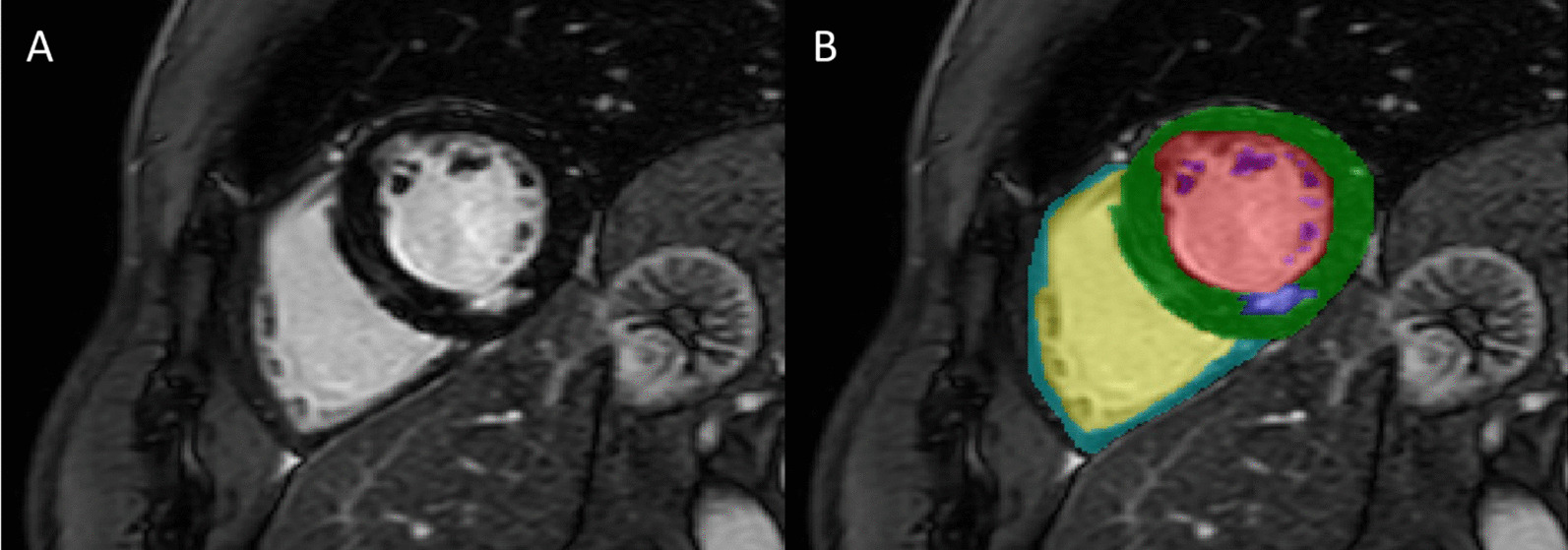
Example of late gadolinium enhanced short axis image with inferior scar (**A**) with labels (**B**). Green—left ventricular myocardium; Cyan—right ventricular myocardium; Dark blue—scar; Purple—papillary muscle; Red—left ventricular blood pool; Yellow—right ventricular blood pool

Studies will undergo structure contouring by a level 3 SCMR trained clinician utilising CVI 42 (Circle Cardiovascular Imaging Inc., Calgary, Alberta, Canada). Second operator labelling will be present. The labelling operator will utilise all available images (eg. short axis, long axis, CINE) to inform the labelling process. The minimum-labelled series will include short-axis CINE and LGE. Labels for CINE images will include the left and right ventricular endo- and epicardium contoured with trabeculations excluded. Papillary muscles and aortic root/ascending aorta will be separately labelled. Scar will be delineated on LGE utilising several methods including manual, full-width half-maximum and n-standard deviations [14] (Fig. 2). Future studies, presentations & publications will be required to declare the utilised segmentation labels. Contours will be transformed to pixel-based annotations utilising specific in-house software.

#### Image quality

As a quality control measure, each full study as well as the individual image slices will receive an image quality score based on a combination of the clinical CMR report and visual assessment by the labelling operator. The quality of the CINE and LGE sequences will be scored using the technical quality comments from the clinical scan report. Additional quality scores will be allocated by the labelling operators according to technical image quality and confidence in the segmentation. This will be used to facilitate clinical interpretability of AI outputs for future clinical use.

#### Model retraining

At least 100 scans containing ischaemic scar will be utilised for the purpose of testing and retraining of a deep learning LGE segmentation model (neural network) developed by our group. For current model retraining, we will extract and reserve a test set (20% of the whole dataset) representing the diversity of data (e.g. variations in scar size, image resolution, age, gender etc.) to validate the final trained AI method. The remaining 80% data will be used for AI model training and development using k-fold cross validation. Evaluation of the retrained model will be undertaken utilising multi-modality assessments including, but not limited to, overlap, distance and volume metrics [5].

#### Public involvement

This study has been supported with regular input from a dedicated Patient and Public Involvement (PPI) group since the inception of the project idea in 2018. The group consists of individuals who have lived with the consequences of ischaemic heart disease and ventricular arrhythmias. The PPI Patient Lead for this taskforce group holds a position on the data access committee for the curated database.

## Discussion

Artificial intelligence research in CMR is being increasingly undertaken with various objectives but model development, generalisation and performance may be hindered by the lack of availability of robust, and representative, training datasets including contrast-enhanced images, multivendor sequences and ICD patients.

The NotIs CMR study aims to collect and curate data from clinically indicated investigations including contrast enhanced CMR imaging with a minimum of 5 years clinical follow up for major adverse cardiovascular events including ventricular arrhythmic events. To our knowledge this will produce the largest disease-specific curated paired LGE-CMR imaging and clinical database with the potential of advancing the field of research utilising AI.

The prospectively collected database allows the potential to integrate patient outcomes (e.g. arrhythmic events) with clinical, imaging and scar characteristics to develop risk prediction models whilst supporting the development of a robust and more broadly clinically applicable AI biventricular scar model to guide future clinical therapies including ventricular tachycardia ablation.

### Supplementary Information

**Additional file 1. **Study and clinical definitions.

## Data Availability

Data sharing and collaboration has a central role in this study. The NotIs CMR study will subsequently make available the data collected for prospective and collaborative research via a Trusted Research Environment at Nottingham University Hospitals NHS Trust. Ethical approval as a research database has been granted for this study, ultimately allowing data sharing through formal request. A study specific data access panel has been organised who will act as guardians of the data. Please contact the corresponding author for an application form and for enquiries. All acquired CMR images and clinical information are available dependent on the study proposal.

## Abbreviations

AI: Artificial intelligence
CMR: Cardiac magnetic resonance imaging
LGE: Late gadolinium enhancement
MACE: Major adverse cardiovascular events
MRI: Magnetic resonance Imaging
