# Supplementary material for: The Nottingham Ischaemic Cardiovascular Magnetic Resonance resource (NotIs CMR): a prospective paired clinical and imaging scar database—protocol
Source: J Cardiovasc Magn Reson. 2023 Nov 27;25:69. doi: 10.1186/s12968-023-00978-1 (PMC10680206; doi:10.1186/s12968-023-00978-1)
Supplement: Supplementary file 1 — Additional file 1. Study and clinical definitions. [file 12968_2023_978_MOESM1_ESM.docx]

The Nottingham Ischaemic Cardiovascular Magnetic Resonance Resource (NotIs CMR) – A Prospective Paired Clinical and Imaging Scar Database: Protocol

Supplementary Information

Definitions

This document contains a list of variables, the definitions thereof and potential sources of data.

Clinical Baseline Variables

All variables are obtained from participants and cross-checked with primary/secondary care documentation. If any discrepancies exist, further information can be sought from the treating clinician/team.

CMR Variables

All variables are obtained from clinical reports and cross-checked with primary/secondary care documentation. If any discrepancies exist, further information can be sought from the treating clinician/team.

Primary Diagnosis:

The primary diagnosis is confirmed only after acquisition of CMR sequences and clinical reporting. Ischaemic heart disease is defined as either (a) presence of ischaemic-pattern LGE in the absence of any other significant cardiac findings felt by the treating clinician to be the primary cardiac disease or (b) alternative imaging confirming the presence of atherosclerotic disease, or a history of previous coronary artery bypass grafting/coronary vessel intervention, in the absence of LGE pattern.

Clinical Outcomes

Obtained from primary/secondary care records with additional information from participants and clinical teams as required.

Mortality:

Data can be obtained from any documented follow up from a healthcare professional and/or automatic NHS spine queries/updates undertaken. Cardiac death is defined as those as a result of a direct result of heart failure, acute coronary syndrome (2018 ESC Fourth Universal Definition) or cardiac arrhythmia (where the arrhythmia is the primary cause of death and not resulting from a non-cardiac cause e.g. electrolyte imbalance). Other options felt to be cardiac in nature will be reviewed and agreed upon by the authors.

Myocardial Infarction

Defined as type 1 myocardial infarction according to the 2018 ESC guidelines “Fourth Universal Definition of myocardial infarction” (section 7.1). Found at: https://academic.oup.com/eurheartj/article/40/3/237/5079081?login=true

Heart Failure Admission

Inpatient admission for which the primary diagnosis is attributed to heart failure. Attendance at A&E does not represent admission unless the outcome is attendance of a heart failure day-case unit for further management of heart failure.

Ventricular Arrhythmia

This is defined as sustained ventricular tachycardia/fibrillation, non-sustained ventricular tachycardia/fibrillation resulting in haemodynamic instability, or non-sustained ventricular tachycardia/fibrillation resulting in cardiac device delivered therapy (anti-tachycardia pacing or shock). Events occurring during/at the presentation of an acute coronary syndrome OR thought to be primarily due to a reversible cause, eg electrolyte imbalance, are NOT included.

Ventricular storm

This is defined as three or more episodes of sustained ventricular arrhythmia occurring with 24hrs requiring either anti-tachycardia pacing or cardioversion/defibrillation, with each event separated by 5 minutes.

Cardiac Device Implantation

Elective or emergency implantation of any pacemaker, implantable cardioverter defibrillator or cardiac resynchronisation device for primary or secondary purpose. Loop recorders and other monitoring devices are excluded.
